# Vaccination with the Surface Proteins MUL_2232 and MUL_3720 of *Mycobacterium ulcerans* Induces Antibodies but Fails to Provide Protection against Buruli Ulcer

**DOI:** 10.1371/journal.pntd.0004431

**Published:** 2016-02-05

**Authors:** Miriam Bolz, Angèle Bénard, Anita M. Dreyer, Sarah Kerber, Andrea Vettiger, Wulf Oehlmann, Mahavir Singh, Malcolm S. Duthie, Gerd Pluschke

**Affiliations:** 1 Swiss Tropical and Public Health Institute, Basel, Switzerland; 2 University of Basel, Basel, Switzerland; 3 Lionex GmbH, Braunschweig, Germany; 4 IDRI, Seattle, Washington, United States of America; University of Tennessee, UNITED STATES

## Abstract

**Background:**

Buruli ulcer, caused by infection with *Mycobacterium ulcerans*, is a chronic ulcerative neglected tropical disease of the skin and subcutaneous tissue that is most prevalent in West African countries. *M*. *ulcerans* produces a cytotoxic macrolide exotoxin called mycolactone, which causes extensive necrosis of infected subcutaneous tissue and the development of characteristic ulcerative lesions with undermined edges. While cellular immune responses are expected to play a key role against early intracellular stages of *M*. *ulcerans* in macrophages, antibody mediated protection might be of major relevance against advanced stages, where bacilli are predominantly found as extracellular clusters.

**Methodology/Principal Findings:**

To assess whether vaccine induced antibodies against surface antigens of *M*. *ulcerans* can protect against Buruli ulcer we formulated two surface vaccine candidate antigens, MUL_2232 and MUL_3720, as recombinant proteins with the synthetic Toll-like receptor 4 agonist glucopyranosyl lipid adjuvant-stable emulsion. The candidate vaccines elicited strong antibody responses without a strong bias towards a T_H_1 type cellular response, as indicated by the IgG2a to IgG1 ratio. Despite the cross-reactivity of the induced antibodies with the native antigens, no significant protection was observed against progression of an experimental *M*. *ulcerans* infection in a mouse footpad challenge model.

**Conclusions:**

Even though vaccine-induced antibodies have the potential to opsonise the extracellular bacilli they do not have a protective effect since infiltrating phagocytes might be killed by mycolactone before reaching the bacteria, as indicated by lack of viable infiltrates in the necrotic infection foci.

## Author Summary

Buruli ulcer is a slow progressing ulcerative disease of the skin and subcutaneous tissue that is most prevalent in West African rural communities. *Mycobacterium ulcerans*, the causative agent of the disease, produces a toxin called mycolactone, which is held responsible for the extensive tissue damage seen in advanced Buruli ulcer lesions. To date, no effective vaccine against the disease exists and it is unclear to what extent antibodies against cell surface antigens of *M*. *ulcerans* play a role in protection. To assess whether vaccine induced antibodies against cell surface proteins can protect against Buruli ulcer, we formulated two surface vaccine candidate antigens, MUL_2232 and MUL_3720, as adjuvanted recombinant proteins and investigated their protective potential in a mouse model of *M*. *ulcerans* infection. Despite the induction of strong antibody responses against the surface molecules and cross-reactivity of the induced antibodies with the antigens in their native context, we did not observe protection against the disease. While the vaccine-induced antibodies could opsonize the extracellular bacilli, infiltrating phagocytes might be killed early by mycolactone.

## Introduction

Buruli ulcer (BU) is a neglected tropical disease of the skin and subcutaneous tissue reported from over 30 countries worldwide. BU is most prevalent in West African countries like Cote d’Ivoire, Cameroon, Benin and Ghana [1,2]. *Mycobacterium ulcerans*, the causative agent of BU, produces a macrolide exotoxin called mycolactone, which is responsible for extensive necrosis of infected subcutaneous tissue leading to the development of large ulcerative lesions, if not treated at an early stage [3]. While extensive surgical removal of the diseased tissue has been the only treatment approach for a long time, since 2004 the WHO recommends eight weeks of combination chemotherapy with rifampicin and streptomycin [4]. This change in treatment strategy has substantially decreased both the amount of surgery required for treatment of extensive skin lesions as well as recurrence rates [5–7]. Nevertheless, BU has remained a huge socioeconomic burden in endemic regions of Africa. Affected populations are typically living in rural regions with limited access to health care services and limited financial resources, frequently resulting in delayed health care seeking and presentations with large ulcerative lesions, which take long time to heal [8,9].

Sero-epidemiological studies have shown that active BU only develops in some of the people exposed to *M*. *ulcerans* [10,11]. Together with reports on spontaneous healing of BU lesions [12,13] and the fact that the risk for young adults to develop BU is much smaller than for children [14], this observation suggests that development of protective immunity against BU is possible [15]. However, it is not clear which immune effector functions are important for protection. Cellular immunity is expected to play a key role in the early intracellular growth phase of *M*. *ulcerans* in macrophages [16–18]. However, induction of T_H_1 responses by vaccination with Bacillus Calmette-Guérin (BCG) or a mycolactone negative *M*. *ulcerans* strain conferred only transient protection in an experimental mouse infection model [19]. Likewise, BCG vaccination seems to lead to cross-reactive immunity to severe forms of BU in clinical trials [20,21], but the BCG mediated induction of cellular response was not able to protect completely from *M*. *ulcerans* disease in either mice or humans [19–21]. In advanced BU lesions, in which clusters of extracellular bacilli dominate, antibodies against surface proteins of *M*. *ulcerans* may be of major importance for conferring protection [18,22,23]. In order to study this hypothesis, two *M*. *ulcerans* surface antigens, MUL_2232 and MUL_3720, were chosen in this study as vaccine candidate antigens. MUL_2232, the 18 kDa small heat shock protein of *M*. *ulcerans*, is an immunodominant cell wall associated protein with a homologue found in *M*. *leprae*, but not in *M*. *bovis* or *M*. *tuberculosis* [10]. MUL_3720 is a 22 kDa molecule with a predicted N-terminal lectin domain and a C-terminal peptidoglycan-binding domain with a putative role in cell attachment and cell-cell interaction [24] that is highly expressed on the surface of the bacilli [25].

Within the framework of a collaborative project (BuruliVac) our goal was to assess whether vaccine induced antibody responses against surface proteins of *M*. *ulcerans* are protective against BU. Here we present immunogenicity studies of MUL_2232 and MUL_3720 formulated as adjuvanted recombinant proteins with Alum, Sigma adjuvant (a squalene oil-in-water emulsion containing Monophosphoryl Lipid A (MPL) and synthetic trehalose dicorynomycolate) or EM048 (glucopyranosyl lipid adjuvant-stable emulsion (GLA-SE) adjuvant system [26,27]). Further, we assessed the potential of the induced immune responses to confer protection against experimental infection in a murine *M*. *ulcerans* infection model.

## Material and Methods

### Ethical statement

All animal experiments performed were approved by the animal welfare committee of the Canton of Basel (authorization number 2375) and the Canton of Vaud (authorization number 2261) and were conducted in compliance with the Swiss animal protection law (Tierschutzgesetz, TSchG, 455). Infection experiments with *M*. *ulcerans* were conducted under Biosafety-level-3 conditions at the École polytechnique fédérale de Lausanne (EPFL).

### Expression and purification of recombinant *M*. *ulcerans* proteins

The potential protein vaccine candidate antigens MUL_2232 (GenBank accession number 4550596) and MUL_3720 (GenBank accession number 4553013) of *M*. *ulcerans* Agy99 were ordered as codon optimized genes for expression in human cells (GenScript) and received in pUC57 plasmids. Expression of the antigens as recombinant proteins in *E*. *coli* was achieved with the pET28a expression system (Novagen, modified to contain an ampicillin selection cassette). Briefly, restriction sites required for further cloning were attached by the use of specifically designed primers for amplification of the codon optimized sequences by polymerase chain reaction (PCR). Primer sequences for MUL_22232 amplification were 5’-TTCCTTCATATGCTGATGAGAACCGACCCTTTTAGA-3’ and 5’-TTCCTTGCGGCCGCTCAAGCCTCAATCACTTCGGGA. Primer sequences for MUL_3720 amplification were 5’-TTCCTTCATATGAGCGATACTCTGACTGAAGGACAG-3’ and 5’-TTCCTTGCGGCCGCGCTCAAGGAATAGTCAGGACCTCT-3’. PCR products were cut by the restriction enzymes NdeI and NotI (New England Biolabs) and subsequently ligated into pET28 to attach an N-terminal 6xHis-tag. After propagation of the generated plasmids in Top10 *E*. *coli* (Invitrogen), control restriction and sequencing of the plasmids ensured selection of appropriate clones for expression of the proteins. Protein expression was induced in *E*. *coli* BL21(DE3) strains (Invitrogen) by addition of 1 mM isopropyl thiogalacoside (Calbiochem) for 4 h at 37°C in lysogeny broth (LB) medium supplemented with Ampicillin. After screening for high level recombinant protein expression by analysis of small induced cultures, larger amounts of recombinant proteins were produced by selected expression clones.

Protein lysates were produced by dilution of the bacterial pellet in PBS, the addition of lysozyme and sonication. After removal of cellular debris by centrifugation, the 6xHis-tagged recombinant proteins were purified by nickel-nitrilotriacetic acetic (Ni-NTA) metal-affinity chromatography. Proteins were eluted with increasing concentrations of imidazole and the integrity and purity of proteins was assessed by SDS-page separation and Coomassie Blue staining (S1 Fig). Final concentrations of the produced recombinant proteins rMUL2232 and rMUL3720 were determined by BCA assay (Pierce) according to the manufacturer’s instructions.

### Adjuvant formulations

Oil-in-Water formulated TLR-4 agonist (GLA-SE, EM048) was produced by the Infectious Disease Research Institute (IDRI) [26,27]. EM048 was mixed with recombinant protein in PBS to a final concentration of 50 μg/ml EM048 and 200 μg/ml recombinant protein. Sigma adjuvant system (Sigma) was reconstituted according to the manufacturer’s instructions and combined with recombinant protein to a final concentration of 200 μg/ml recombinant protein. Imject Alum (Thermo Scientific) was mixed with recombinant protein according to the manufacturer’s instructions to a final volume ratio of Imject Alum to immunogen of 1:1 and a final concentration of 200 μg/ml recombinant protein.

### Immunization of mice

Immunogenicity of the described vaccine formulations was studied in 8 week old female BALB/c mice (Janvier). Groups of five mice were immunized three times by the subcutaneous (s.c.) route in the scruff of the neck with 100 μl of the adjuvanted proteins in three week intervals. Prior to the first immunization as well as before every new immunization mice were bled by the tail vein and serum gained by centrifugation of the blood in SST Microtainer tubes (Becton, Dickinson and Company). An additional blood collection was performed three weeks and six months after the last immunization.

### Enzyme-linked immunosorbent assays (ELISA)

Serum immunoglobulin G (IgG) antibody titers were determined by ELISA on recombinant protein with all incubation steps performed at room temperature (RT). 10 μg/ml of rMUL2232 or rMUL3720, respectively, were coated on ELISA plates (Maxisorp; Nunc) by incubation overnight. After blocking with 5% skim milk/PBS for 1 hour, plates were incubated with dilution series of sera from immunized mice in 0.5% skim milk/PBS for two hours, washed and incubated with alkaline phosphatase-conjugated goat anti-mouse monoclonal antibody (mAb; Sigma) as secondary antibodies for 1 hour. Plates were washed prior to development with *p*-nitrophenyl phosphate (Sigma) as substrate. The optical density (OD) of the reaction product was measured at 405 nm with a microplate absorbance reader (Sunrise Absorbance Reader; Tecan). The threshold for endpoint titer determination was defined as the double of the mean measurements plus the mean standard deviation of a dilution series done without primary antibody and a dilution series done with pre-bleed serum. Individual serum dilution series were approximated with sigmoidal dose-response curves and the reciprocal dilution of the intersection between the curve and the threshold was defined as individual endpoint titer (GraphPad Prism software, Graph-Pad Software Inc.).

For the determination of IgG subclasses, ELISA was performed as described above with the use of subclass specific alkaline phosphate-conjugated secondary antibodies (Southern Biotech).

### Western blot analysis

According to the manufacturer’s instructions, 10 μg of *M*. *ulcerans* whole cell lysate was loaded on a prefabricated 4–12% gradient gel (NuPAGE Novex 4–12% Bis-Tris Gel; Invitrogen) with MES running buffer under reducing conditions. A dry-blotting system (iBlot; Invitrogen) was used to electrophoretically transfer the separated proteins to nitrocellulose membranes, which were subsequently blocked in 5% skim milk/PBS over night at 4°C. Membranes were cut into strips and individually incubated with appropriate dilutions of serum of immunized mice in 1% skim milk/0.05% Tween20 / PBS for 1 hour at RT. After several washing steps in 1% skim milk/0.05% Tween20/PBS, a HRP-conjugated goat anti-mouse IgG γ-chain mAb (Southern Biotech) was used as secondary antibody and incubated for 1 hour at RT. Excess antibody and skim milk residuals were washed away with PBS and blots were then developed using ECL Western blotting detection reagents (ECL Western blotting Substrate; Pierce).

For the determination of the Western blot endpoint titers, individual dilution series of sera were processed as described above. Development of the entire set of strips by the ECL system was done on one single film. Development time was chosen as the shortest time needed for detecting a specific signal in at least one strip for every serum dilution, e.g. lowest dilution, and for every dilution series of individual sera at least one strip with no specific signal, e.g. highest dilution. Western blot endpoint titers were defined as the reciprocal value of the last dilution that yielded a specific band in Western blotting on the film.

### Immunofluorescence assays on paraffin embedded *M*. *ulcerans*

Immunofluorescence assays with sera of immunized mice on paraffin embedded *M*. *ulcerans* bacteria were performed as previously described [25]. Briefly, African *M*. *ulcerans* isolates were embedded into paraffin, cut into 3 μm thin sections and mounted on Superforst Plus glass slides (Thermo Scientific). Sections were then deparaffinised, rehydrated and pre-treated with 1mM EDTA buffer pH = 8 for epitope retrieval as described for tissue sections in immunohistochemistry [28]. Unspecific binding was prevented by incubation of the bacteria in 1.5% goat serum in PBS for 1 hour at RT. Appropriately diluted mouse mAbs specific for MUL_2232 and MUL_3720 were used as primary antibodies. Detection of the specific binding of primary antibodies was done with an Alexa488 labelled secondary goat anti-mouse total IgG (H+L) antibodies (Life Technologies). Image acquisition was performed on a confocal laser microscope (Carl Zeiss, Axiovert 200M).

### Active immune protection experiments in mice

Active immune protection experiments were conducted with groups of eight female 8 weeks old BALB/c mice. Mice were immunized twice s.c. in the scruff of the neck with 100 μl of the adjuvanted proteins in three week intervals. Three weeks after the second immunization and prior to infection with *M*. *ulcerans*, mice were bled by the tail vein and successful immunization was verified by testing the sera for specific antibodies in ELISA and Western blotting as described above. All *M*. *ulcerans* infection experiments were conducted under BSL-3 conditions.

The *M*. *ulcerans* strain S1013 used for the experimental infection of mice was isolated in 2010 from the ulcerative lesion of a Cameroonian BU patient [29]. Bacteria were cultivated in BacT/ALERT medium (Biomerieux) for six weeks, recovered by centrifugation and diluted in sterile PBS to 125 mg/ml wet weight. Mice were infected with 1.5 x 10^6^ (high dose) or 1.5 x 10^5^ (low dose) bacteria in PBS into the left hind foot pad three weeks after the last immunization.

The course of the infection was followed by weekly measurements of the foot pad thickness with a caliper. At days 63 (high dose) and 87 (low dose) after experimental infection, mice were euthanised, blood samples harvested through cardiac puncture and foot pads aseptically removed for enumeration of *M*. *ulcerans* bacteria or histopathology. Mouse foot pads designated for enumeration of *M*. *ulcerans* bacteria were dipped in 70% ethanol, dried under the laminar flow, cut into four pieces with a scalpel and transferred to reinforced hard tissue grinding tubes (MK28-R, Precellys) containing 750 μl of BacT/ALERT medium. Tissue homogenization was performed with a Precellys 24-Dual tissue homogenizer (3 x 20 s at 5000 rpm with 30 s break), the lysate was transferred to a clean tube and the lysis tube still containing tissue residuals refilled with additional 750 μl of BacT/ALERT medium. The remains were homogenized a second time as described above and the individual two lysates were pooled [30].

DNA from 100 μl of a 1:50 dilution of the foot pad lysate in PBS was extracted as described by Lavender and Fyfe [31]. Extracted DNA was then analysed for insertion sequence (IS) 2404 by quantitative (q) PCR as previously described [31]. For graphic representation of the results, cycle threshold (Ct) values were converted into genome copy numbers per foot pad by applying a standard curve established for IS2404 by *Fyfe et al*. [32].

### Histopathology

Mouse foot pads designated for histopathological analysis were removed above the ankle and immediately transferred to 10% neutral-buffered formalin solution (approx. 4% formaldehyde, Sigma) for fixation during 24 hours at room temperature. Subsequently, the foot pads were decalcified in 0.6 M EDTA and 0.25 M citric acid for 12 days at 37°C and transferred to 70% ethanol for storage and transport. Foot pad samples were dehydrated and embedded into paraffin. 5 μm thin sections were cut, deparaffinised, rehydrated, and stained with Haematoxylin/Eosin (HE, Sigma, J.T. Baker) or Ziehl-Neelsen/Methylene blue (ZN, Sigma) according to WHO standard protocols [33]. Stained sections were mounted with Eukitt mounting medium (Fluka). Pictures were taken with a Leica DM2500B microscope or with an Aperio scanner.

## Results

### GLA-SE adjuvanted recombinant *M*. *ulcerans* protein formulations elicited specific antibody responses

**Fig 1:**
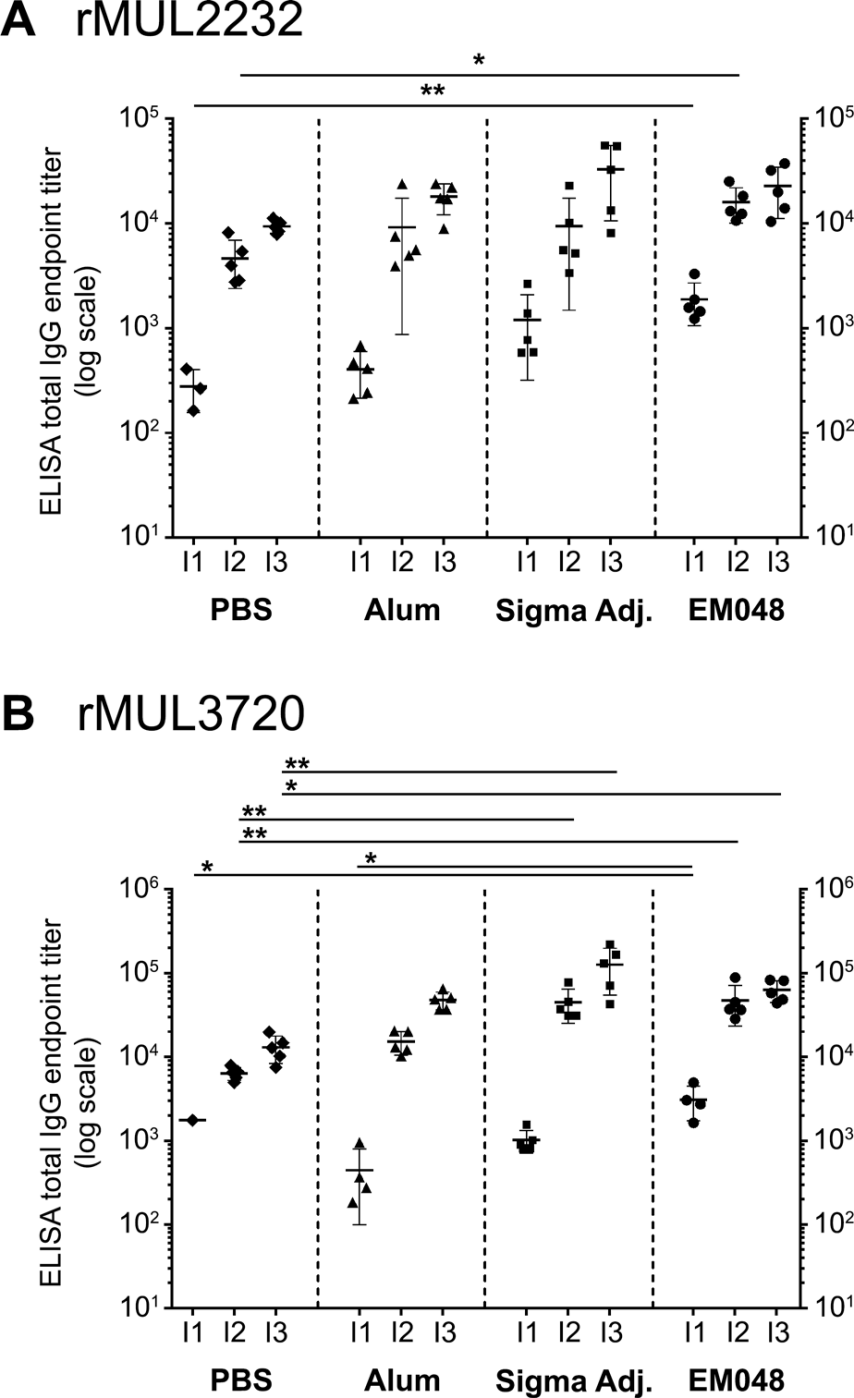
Immunogenicity of recombinant protein/EM048 formulation in comparison to commercially available adjuvants for mice. Groups of five BALB/c mice were immunized three times in three week intervals with 20 μg of rMUL2232 (A) or rMUL3720 (B) in PBS or formulated with Alum, Sigma Adjuvant or EM048. Serum three weeks after every immunization (I1, I2 and I3) was analysed in ELISA on the respective recombinant protein. Depicted are individual endpoint IgG titers as determined in one single ELISA, the mean (line) ± standard deviation. Values that are zero are not depicted but were included for statistical analysis. Overall statistical significance was calculated by Kruskal-Wallis test per immunization time point (A: P_I1_ = 0.0018, P_I2_ = 0.0372 and P_I3_ = ns.; B: P_I1_ = 0.0095, P_I2_ = 0.0011, P_I3_ = 0.0041.). Individual statistical differences between groups were assessed by the Dunn procedure and are depicted if detected (* *p* ≤ 0.05; ** *p* ≤ 0.01).

**Fig 2:**
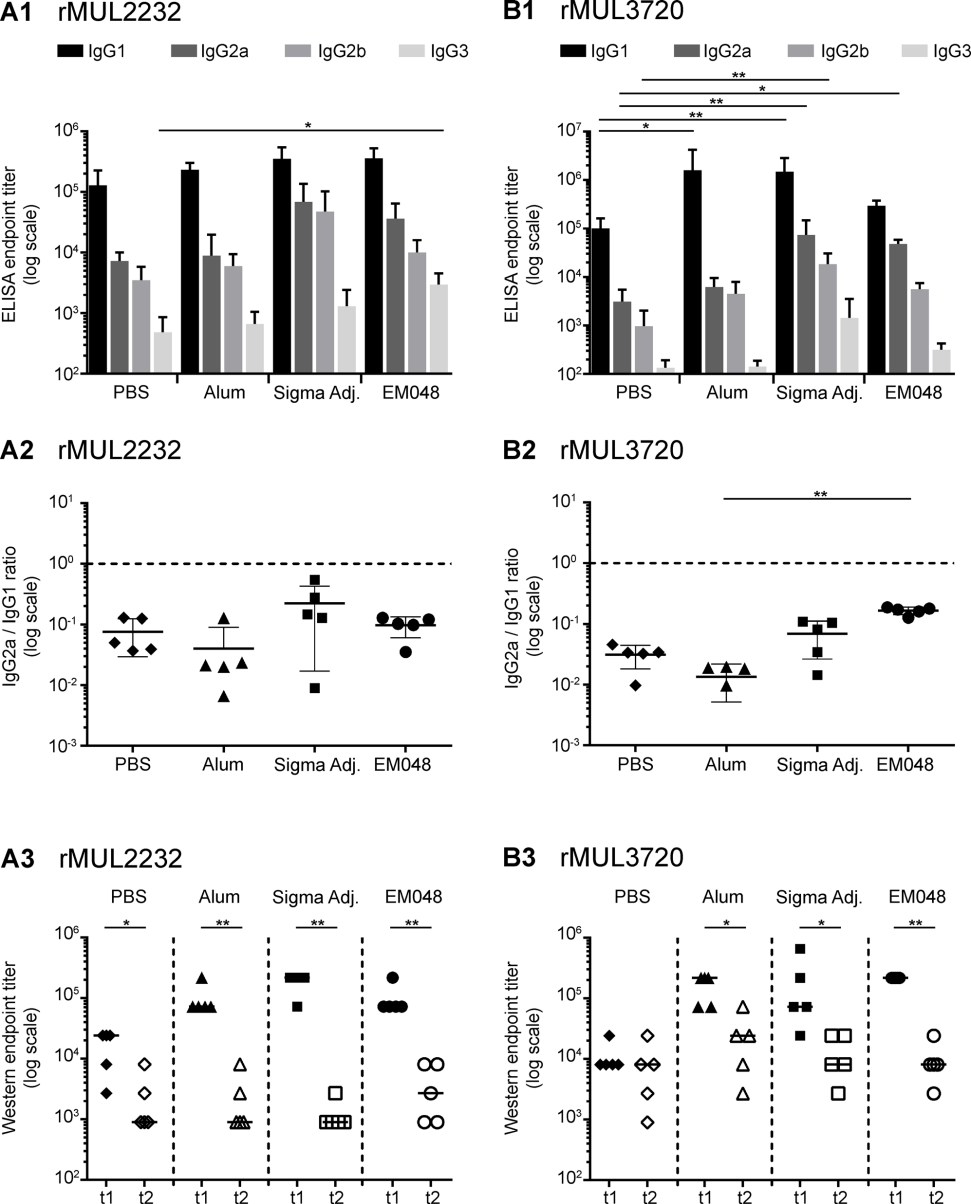
Assessment of Immunoglobulin G subclasses and the longevity of antibody responses induced by immunization. Immunoglobulin G (IgG) subclasses were determined in serum collected three weeks after the third immunization with rMUL2232 (A1) and the indicated adjuvant or rMUL3720 (B1) and the indicated adjuvant. ELISA on recombinant protein was performed with secondary antibodies specific for four IgG subclasses: IgG1 (black), IgG2a (dark grey), IgG2b (light grey), IgG3 (very light grey). Depicted are the mean individual endpoint IgG titers determined in one single ELISA (bar), and the standard deviation (error bar). Overall statistical significance was calculated by Kruskal-Wallis test (A1: P_IgG1_ = 0.0265., P_IgG2a_ = 0.0205, P_IgG2b_ = ns., P_IgG3_ = 0.0276; B1: P_IgG1_ = 0.0034., P_IgG2a_ = 0.0017, P_IgG2b_ = 0.0048, P_IgG3_ = ns.). Individual statistical differences between groups were assessed by the Dunn procedure and are depicted if detected (* *p* ≤ 0.05; ** *p* ≤ 0.01). The ratio of IgG2a to IgG1 was determined for individual animals accordingly (A2 and B2). Depicted are the individual values, the mean IgG2a to IgG1 ratio (bar) and the standard deviation (error bar). Overall statistical significance was calculated by Kruskal-Wallis test (A2: P = ns.; B2: P = 0.0024). Individual statistical differences between groups were assessed by the Dunn procedure and are depicted if detected (** *p* ≤ 0.01). Total IgG titers in individual immunized mice three weeks (t1) and six months (t2) after the third immunization were compared by Western blotting on *M*. *ulcerans* lysate. Depicted are individual Western blot endpoint titers and the median per group (line) for rMUL2232 immunized animals (A3) and rMUL3720 immunized animals (B3). Statistical significance was assessed by Mann-Whitney test and is depicted if detected (* *p* ≤ 0.05; ** *p* ≤ 0.01).

The two *M*. *ulcerans* vaccine candidate antigens MUL_2232 and MUL_3720 were expressed as 6xHis-tagged recombinant proteins in *E*. *coli* and purified via a Ni-NTA column (S1 Fig). Mice were immunized three times with either 20 μg of MUL_2232 or MUL_3720 formulated with the human-compatible GLA-SE adjuvant EM048. Alum and Sigma adjuvant were used as control adjuvants. ELISA with mouse sera on the respective recombinant proteins showed that all formulations elicited robust antigen specific serum IgG responses (Fig 1) dominated by IgG1 and only a minor proportion of IgG3 (Fig 2A1 and 2B1).

While rMUL2232-specific IgG2a to IgG1 ratios showed no significant differences among the different formulations tested (Fig 2A2), IgG2a to IgG1 ratio was significantly higher when mice were immunized with rMUL3720 EM048 adjuvanted candidate vaccine than with the two other formulations (Fig 2B2).

**Fig 3:**
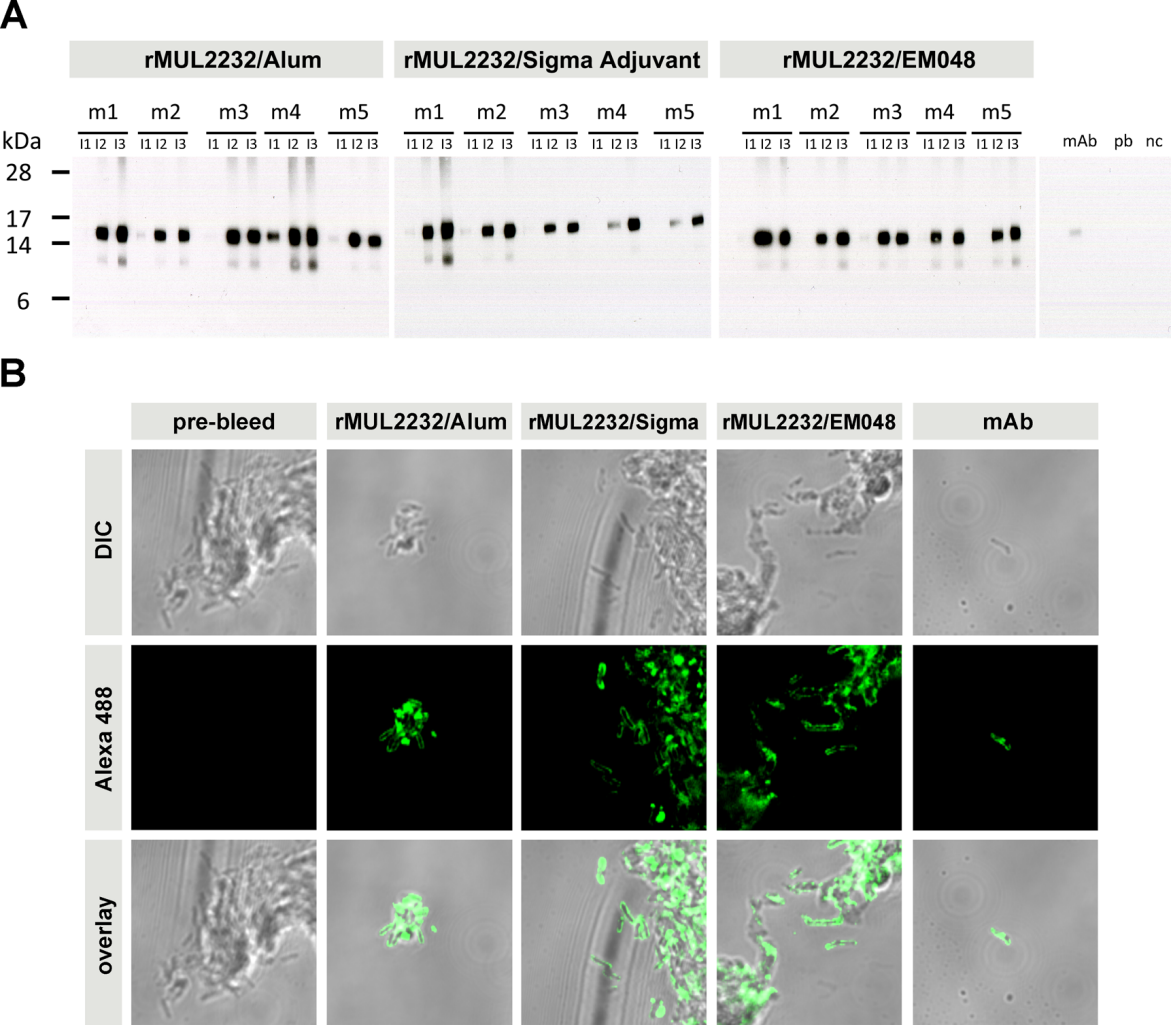
Cross reactivity of the immune sera with *M*. *ulcerans*. (A) Groups of five BALB/c mice (m1 –m5) were immunized three times in three week intervals with 20 μg of rMUL2232 formulated with Alum, Sigma Adjuvant or EM048. Serum three weeks after every immunization (I1, I2 and I3) was analysed by Western blotting on *M*. *ulcerans* lysate. Monoclonal anti-MUL_2232 antibody (mAb) served as positive control, pre-bleed (pb) serum or no primary antibody (nc) as negative controls. (B) Sera from three weeks after the third immunization with rMUL2232 and indicated adjuvant were used for indirect immunofluorescence staining on paraffin embedded *M*. *ulcerans* bacteria with an Alexa488 labelled secondary antibody. Pre-bleed serum did not stain the bacteria. Sera of immunized mice (a mix of sera from five individual mice per group) did reveal surface staining similar to the staining achieved with anti-MUL_2232 monoclonal antibody (mAb).

Western blotting analyses of sera against *M*. *ulcerans* lysates showed specific bands of the expected molecular weight of MUL2232 and MUL3720 (Fig 3A, S2A Fig). Sera of immunized mice also recognized *M*. *ulcerans* bacteria in an indirect immunofluorescence assay (IFA) performed on paraffin embedded *M*. *ulcerans* bacteria (Fig 3B, S2B Fig). For both target antigens the previously demonstrated surface localization [25] was confirmed. Six months after the last immunization, antibody responses in all groups of immunized mice had dropped significantly. However, specific antibody titres were still higher than in the pre-immune sera and sufficient to elicit signals in Western blotting analyses (Fig 2A3 and 2B3).

### Assessment of protectivity of the vaccine induced antibody responses in an experimental *M*. *ulcerans* infection mouse model

**Fig 4:**
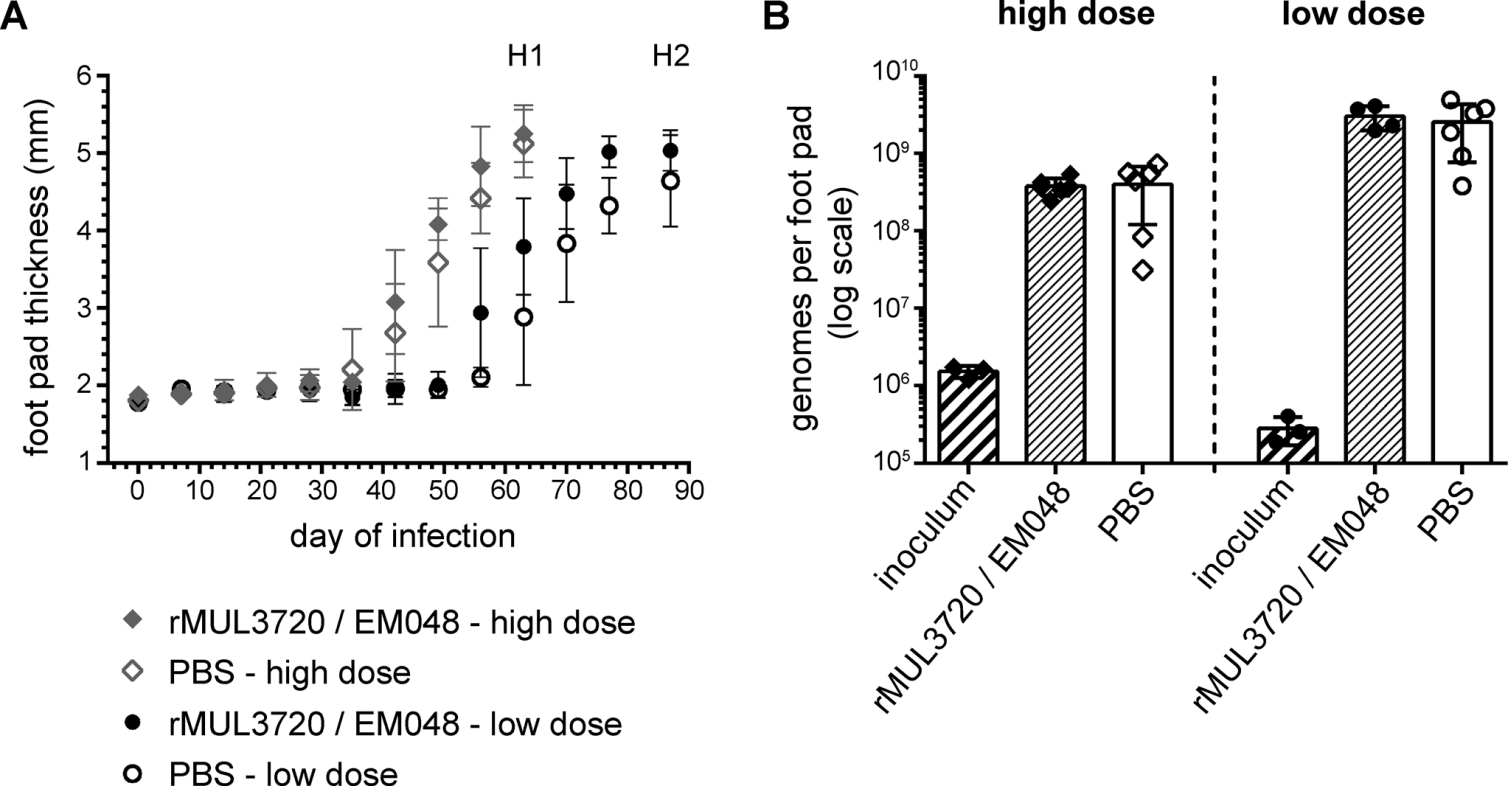
Evaluation of the protective potential of immunization with rMUL3720/EM048 formulation in a *M*. *ulcerans* infection mouse model. Groups of eight BALB/c mice were immunized twice with 20 μg of rMUL3720/EM048 or PBS alone as infection control. Three weeks after the last immunization mice were challenged with a high dose or a low dose of *M*. *ulcerans* (inoculum) into the left hind foot pad. Infection was followed by measuring foot pad thickness with a caliper (A) until mice were euthanized at day 63 after infection (high dose, H1) or at day 87 after infection (low dose, H2). Depicted is the mean foot pad thickness (diamond/circle) ± standard deviation of the differently immunized groups. (B) Bacterial load in infected foot pads was determined by qPCR for six mice per group. Depicted are individual measurements as genome copies per foot pad, the mean (line) ± standard deviation.

Given that antigens formulated with the human-compatible EM048 elicited higher total IgG responses after two injections and with similar IgG1, but higher IgG2a, IgG2b, IgG3 antibody levels compared to formulation with Alum, we determined the protective potential of these vaccine formulations in an experimental *M*. *ulcerans* infection mouse model. Because the increase of total IgG titers after a third immunization was not significant (Fig 1), groups of eight mice were immunized twice with rMUL3720/EM048. Three weeks after the second immunization mice were infected into the left hind foot pad with an inoculum of 1.5 x 10^6^ (high dose) or 1.5 x 10^5^ (low dose) of *M*. *ulcerans* bacilli. The course of the infection was followed by weekly measurements of the foot pad thickness with a caliper. Mice in all groups infected with the high dose of bacteria showed first signs of inflammation and foot pad swelling seven weeks after infection (Fig 4A). Swelling gradually increased over time, until mice had to be euthanised at day 63 and the bacterial load was determined by qPCR (Fig 4B). Compared to the amount of *M*. *ulcerans* DNA that was contained in the inoculum, a roughly 250 times increase had occurred both in immunized and control animals during the 63 days of infection (Fig 4B).

**Fig 5:**
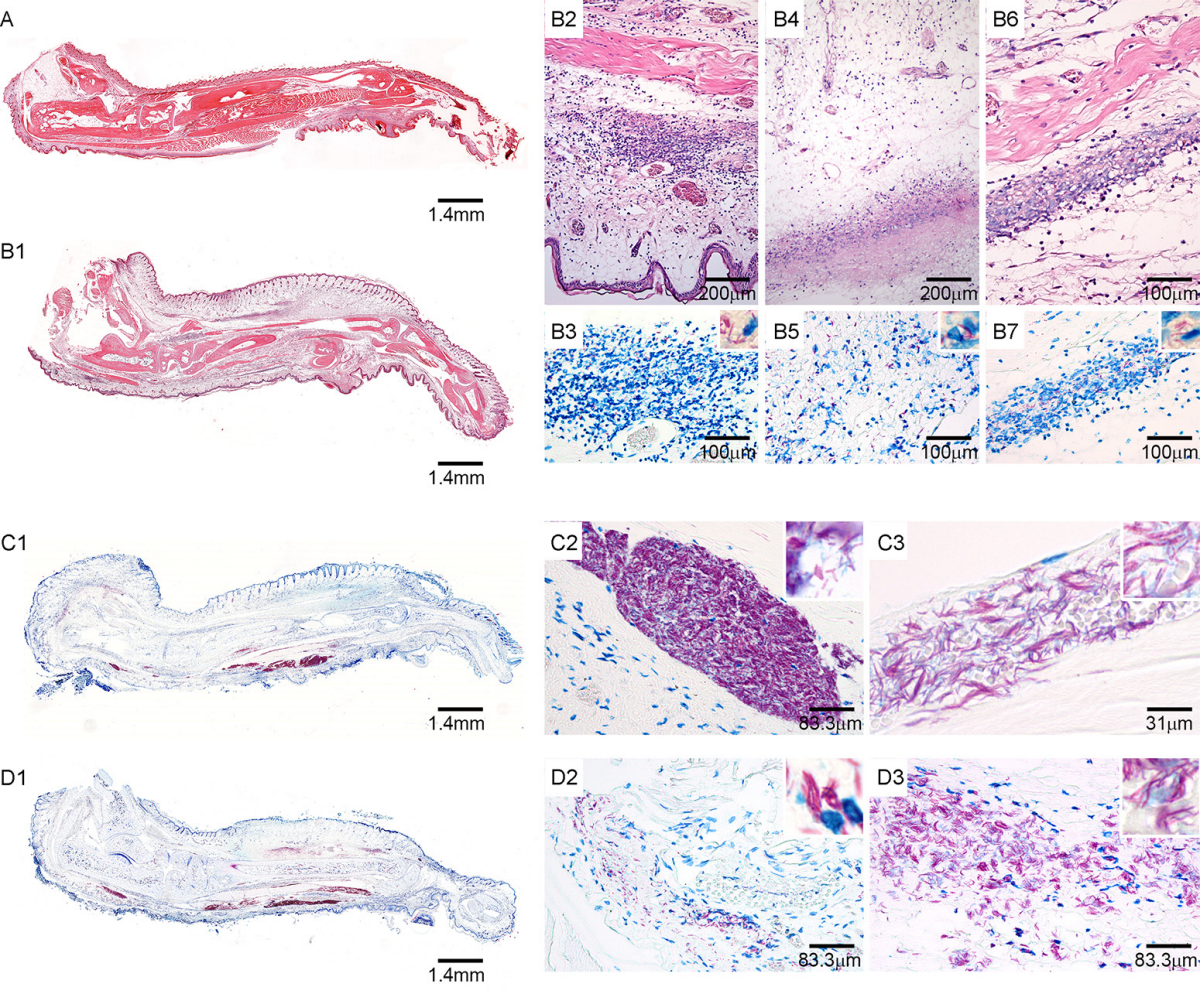
Histopathological evaluation of mouse footpads following *M*. *ulcerans* infection. Histological sections of foot pads from *M*. *ulcerans*-infected mice were stained with Haematoxylin/Eosin (A, B1—B7) or Ziehl-Neelsen/Methylene blue (ZN) (C1—C4, D1—D4). Mice challenged with a high dose of *M*. *ulcerans* developed typical signs of infection in the mouse foot pad model until day 63 after infection. Compared to a control foot pad (A) the infected foot pad of a representative immunized mouse (B1) showed necrosis and infiltration interspersed with AFB at the ankle (B2, B3) as well as at the base of the foot (B6, B7). Oedema was marked on top of the foot pad (B4) where bacteria were also found (B5). Immunized mice (C1) as well as control mice (D1) challenged with a lower dose of *M*. *ulcerans* developed strong infection foci until day 87 after infection. AFB appeared as big, dense clumps (C2) or in close association with infiltrating cells (D2) towards the heel of the infected foot pads as well as in the middle of the foot (C1, D1). AFB were also present in oedematous tissue in the upper half of the foot (D3) and appeared in filamentous organization (C3).

Histopathological analysis of representative foot pads revealed the presence of oedema (Fig 5B1 and 5B4) and slight infiltration at the site of infection (Fig 5B6) as well as at the heel of the foot pad (Fig 5B2). Acid fast bacilli (AFB) were found at all sites where infiltration occurred (Fig 5B3, 5B5 and 5B7).

In mice infected with the low dose inoculum, foot pad swelling started seven to eight weeks after infection (Fig 4A) and the increase in *M*. *ulcerans* DNA content was about 9500 fold in 87 days. Also with the lower challenge dose no difference in bacterial load was observed between immunized and control immunized animals (Fig 4B). Large clumps of AFB were found in all infected foot pads irrespective of the immunization status of the mice (Fig 5C1, 5C2 and 5D1. AFB occurred in clumps (Fig 5C2), organized within filamentous structures principally located in oedematous tissue (Fig 5C3 and 5D3) and in close contact with infiltrating cells (Fig 5D2). Similarly, rMUL2232/EM048 did not induce any protective effect (S3 Fig).

### *M*. *ulcerans* infection failed to boost immunization-induced antibody responses

**Fig 6:**
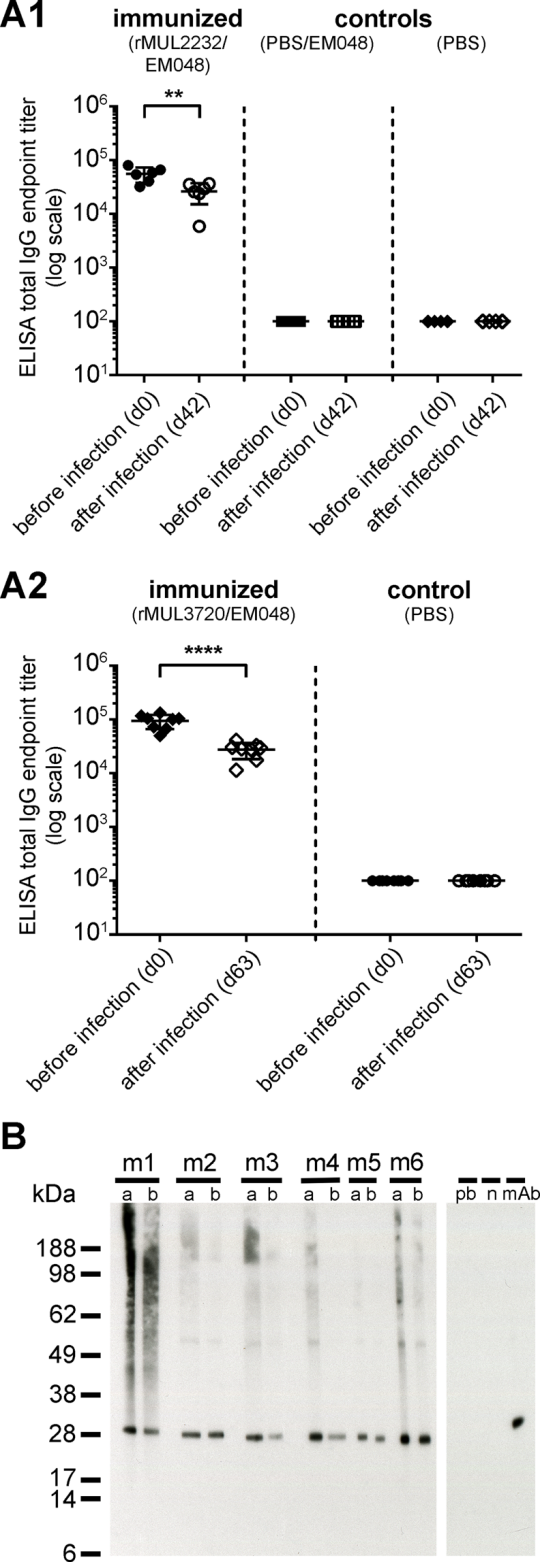
Evaluation of the booster effect of *M*. *ulcerans* infection on the pre-existing antibody response in rMUL3720/EM048 immunized and subsequently infected mice. (A1) Serum of six BALB/c mice immunized with rMUL2232/EM048 prior to infection (before infection) with *M*. *ulcerans* was compared to serum of the same animals after 42 days of infection (after infection) in ELISA on rMUL2232. Control immunized animals had received PBS/EM048 or only PBS prior to infection. Depicted are individual endpoint IgG titers as determined in one single ELISA, the group mean (line) ± standard deviation. Statistical significance was calculated by the Student’s t- test (** *p* ≤ 0.01). (A2) Serum of eight BALB/c mice immunized with rMUL3720/EM048 prior to infection with *M*. *ulcerans* was compared to serum of the same animals 63 days after infection with *M*. *ulcerans* bacteria in ELISA on rMUL3720. Control immunized animals (control) had only received PBS prior to infection. Depicted are individual endpoint IgG titers as determined in one single ELISA, the mean (line) ± standard deviation. Statistical significance was calculated by the Student’s t- test (**** *p* ≤ 0.0001). (B) Serum of six BALB/c mice (m1 –m6) immunized with rMUL3720/EM048 prior to infection with *M*. *ulcerans* (a) was compared to serum of the same animals after 42 days of infection (b) by Western blotting on *M*. *ulcerans* whole cell lysate. Monoclonal anti-MUL_3720 antibody (mAb) served as positive control, pre-bleed (pb) serum or no primary antibody (nc) as negative controls.

Antibody titers in immunized mice before infection (S4 Fig) and after 63 days of infection were directly compared to elucidate whether the immunization-induced antibody responses were increased by exposure to the target antigens in the native context of the infecting bacilli. No booster effect was observed after infection with the high dose inoculum of *M*. *ulcerans* (Fig 6A1 and 6A2). On the contrary, both ELISA and Western blotting analyses demonstrated a significant drop in specific antibody titres after infection (Fig 6B), which represents most probably a natural decrease over time. Furthermore, neither non-immunized nor control-immunized animals raised a specific antibody response against rMUL2232 (Fig 6A1) or rMUL3720 (Fig 6A2) and Western blotting analyses of these control sera revealed a profound lack of any *M*. *ulcerans* specific antibody responses in the course of infection (Fig 6B).

## Discussion

Currently, there is no highly effective vaccine against the mycobacterial diseases tuberculosis, leprosy and BU available. BCG was originally developed as a vaccine against tuberculosis, but, dependent on the study site, a protective efficacy ranging from 20% to 90% was also observed against leprosy [34,35]. Similarly, BCG was found to offer a short-lasting protection of 47% against BU in a controlled clinical trial in Uganda [20], reconfirming results of a previous smaller trial [36]. However, case-control studies have failed to provide evidence of a lasting protective effect of routine BCG vaccination against BU [37–39]. Yet in *M*. *ulcerans* mouse infection models any other vaccine candidate has so far outperformed the effectiveness of BCG [18].

As a partner in the collaborative research project BuruliVac, we seeked to assess the potential to develop a protein based subunit vaccine against BU. Here we report results obtained with two cell surface exposed *M*. *ulcerans* proteins formulated with the adjuvant EM048. In spite of the development of robust humoral immune responses, none of the vaccination formulations tested conferred protection in the experimental *M*. *ulcerans* mouse foot pad infection model.

Choosing potential protective antigens for the inclusion into a protein subunit vaccine against BU was difficult, since the nature of protective immune responses against the disease is unclear. Given the mainly extracellular location of *M*. *ulcerans* in advanced lesions, the choice of surface exposed proteins seemed most attractive. Additionally, *M*. *ulcerans* specific proteins were of preference, because it was already observed that proteins from the closely related pathogens *M*. *bovis* and *M*. *leprae* were not very effective in conferring cross-protectivity, despite high sequence homology [40–42]. We have chosen MUL_2232 for its known surface localization, its strong immunogenicity and its missing homolog in *M*. *tuberculosis* [10]. The second candidate, MUL_3720, was identified in a screen for potential diagnostic antigens conducted in our laboratory [24]. Homologs of MUL_3720 are absent in other mycobacterial pathogens prevalent in BU endemic areas. Furthermore, it is highly expressed and most importantly it is located on the surface of *M*. *ulcerans* [25].

In light of current literature available on protective immunity to BU we have chosen to formulate the selected antigens with the human compatible adjuvant GLA-SE. GLA is a synthetic Toll-like receptor 4 agonist, that has been demonstrated to confer potent adjuvant activity for various antigens [26,43–45] when formulated with a squalene based oil-in-water stable emulsion (SE). While SE is an adjuvant on its own, the addition of GLA biases the induced cell mediated immunity (CMI) toward a T_H_1 type immune response, an observation made for several antigens tested so far [43,46]. However, formulations of the recombinant proteins rMUL2232 and rMUL3720 with EM048, the specific GLA-SE adjuvant used in this study, did not lead to such a clear shift of CMI towards T_H_1, as we have assessed by the IgG2a/IgG1 ratios. Yet compared to Alum, which is still the adjuvant most commonly used in human vaccines, EM048 induced significantly higher antibody titres with both recombinant proteins investigated. All mice immunized with adjuvanted recombinant protein vaccine-candidates and later challenged with *M*. *ulcerans* had mounted strong specific antibody responses, which were cross-reactive with the antigens in the native context on the bacterial cell surface. Nevertheless, these mice were not protected from disease. Opsonisation of the bacteria by the immunization-induced antibodies may not lead to protection, since the clusters of the extracellular *M*. *ulcerans* found in advanced BU lesions are imbedded in necrotic tissue. Infiltrating macrophages are therefore not able to reach the bacteria, since infiltrating cells seem to be killed by mycolactone before reaching the infection foci. On the other hand, it is not completely ruled out that antibodies against other target structures may be protective. Neutralizing antibodies against the poorly immunogenic macrolide toxin mycolactone for example could potentially confer protection against disease. The fact that the response of immunized mice was not boosted upon progressive infection with *M*. *ulcerans* could hint to another obstacle for vaccine development against BU. The apparent general lack of antibody responses in non-immunized challenged control mice that was observed by us and others [23] is surprising, and requires further investigation. A number of studies have reported systemic T-cell anergy in patients with BU, but development of antibody responses against *M*. *ulcerans* was detected in a majority of patients [10,47–49].

In the framework of this study we have developed methods to evaluate the protective capacity of candidate vaccines in the *M*. *ulcerans* mouse footpad infection model. The fact that we did not see protection in immunized animals could have many explanations; including insufficient bias towards a T_H_1 type of CMI. Most likely immunization with only one antigen is generally not sufficient for protection against disease. Considering that vaccination with a mycolactone deficient mutant strain of *M*. *ulcerans* did not lead to full protection in the mouse model [19], the development of a multivalent subunit vaccine may be the right strategy to pursue.

## Supporting Information

S1 Fig Two *M*. *ulcerans* candidate vaccine antigens expressed as recombinant proteins in *E*. *coli*. Indicated amounts of rMUL2232 (A) or rMUL3720 (B) were resolved on SDS-page and stained with Coomassie blue.
(PDF)

S2 Fig Cross reactivity of immune sera with *M*. *ulcerans*. (A) Groups of five BALB/c mice (m1 –m5) were immunized three times in three week intervals with 20 μg of rMUL3720 formulated with Alum, Sigma Adjuvant or EM048. Serum after every immunization (I1, I2 and I3) was analysed by Western blotting on *M*. *ulcerans* lysate. Monoclonal anti-MUL_3720 antibody (mAb) served as positive control, pre-bleed (pb) serum or no primary antibody (nc) as negative controls. (B) Sera from three weeks after the third immunization with rMUL3720 and indicated adjuvant were used for indirect immunofluorescence staining on paraffin embedded *M*. *ulcerans* bacteria with an Alexa488 labelled secondary antibody. Pre-bleed serum did not stain the bacteria. Sera of immunized mice (a mix of sera from five individual mice per group) did reveal surface staining similar to the staining achieved with anti-MUL_3720 monoclonal antibody (mAb).
(PDF)

S3 Fig Evaluation of the protective potential of immunization with rMUL2232/EM048 formulation in a *M*. *ulcerans* infection mouse model. Groups of six BALB/c mice were immunized twice with 20 μg of rMUL2232/EM048, PBS/EM048 or PBS alone as infection control. Three weeks after the last immunization mice were challenged with *M*. *ulcerans* (inoculum) into the left hind foot pad. Infection was followed by measuring foot pad thickness with a caliper (A1) until mice were euthanized at day 42 after infection. Depicted is the mean foot pad thickness (diamond/dot) ± standard deviation of the differently immunized groups. (A2) Bacterial load in infected foot pads was determined by qPCR for five mice per group. Depicted are individual measurements as genome copies per foot pad, the mean (line) ± standard deviation.
(PDF)

S4 Fig Reactivity of immune sera on *M*. *ulcerans* lysate. Groups of eight BALB/c mice were immunized twice with 20 μg of rMUL3720/EM048 or PBS only as infection control. Serum prior to infection with *M*. *ulcerans* was analysed by Western blotting on *M*.*ulcerans* lysate. Monoclonal anti-MUL_3720 antibody (mAb) served as positive control, pre-bleed (pb) serum or no primary antibody (neg) as negative controls. C1 and C2 each represent a mix of sera of eight mice immunized with PBS only.
(PDF)
