# Supplementary material for: Vaccination with the Surface Proteins MUL_2232 and MUL_3720 of Mycobacterium ulcerans Induces Antibodies but Fails to Provide Protection against Buruli Ulcer
Source: PLoS Negl Trop Dis. 2016 Feb 5;10(2):e0004431. doi: 10.1371/journal.pntd.0004431 (PMC4746116; doi:10.1371/journal.pntd.0004431)
Supplement: S1 Fig — Indicated amounts of rMUL2232 (A) or rMUL3720 (B) were resolved on SDS-page and stained with Coomassie blue. (PDF) [file pntd.0004431.s001.pdf]

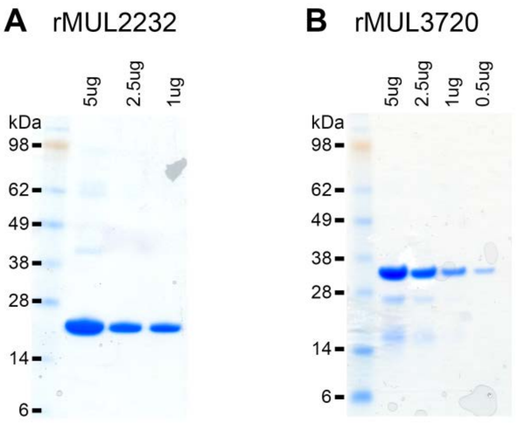

Figure S1: Two *M. ulcerans* candidate vaccine antigens expressed as recombinant proteins in *E. coli*.

Indicated amounts of rMUL2232 (A) or rMUL3720 (B) were resolved on SDS-page and stained with Coomassie blue.
