# Supplementary material for: Vaccination with the Surface Proteins MUL_2232 and MUL_3720 of Mycobacterium ulcerans Induces Antibodies but Fails to Provide Protection against Buruli Ulcer
Source: PLoS Negl Trop Dis. 2016 Feb 5;10(2):e0004431. doi: 10.1371/journal.pntd.0004431 (PMC4746116; doi:10.1371/journal.pntd.0004431)
Supplement: S2 Fig — (A) Groups of five BALB/c mice (m1 –m5) were immunized three times in three week intervals with 20 μg of rMUL3720 formulated with Alum, Sigma Adjuvant or EM048. Serum after every immunization (I1, I2 and I3) was analysed by Western blotting on M. ulcerans lysate. Monoclonal anti-MUL_3720 antibody (mAb) served as positive control, pre-bleed (pb) serum or no primary antibody (nc) as negative controls. (B) Sera from three weeks after the third immunization with rMUL3720 and indicated adjuvant were used for indirect immunofluorescence staining on paraffin embedded M. ulcerans bacteria with an Alexa488 labelled secondary antibody. Pre-bleed serum did not stain the bacteria. Sera of immunized mice (a mix of sera from five individual mice per group) did reveal surface staining similar to the staining achieved with anti-MUL_3720 monoclonal antibody (mAb). (PDF) [file pntd.0004431.s002.pdf]

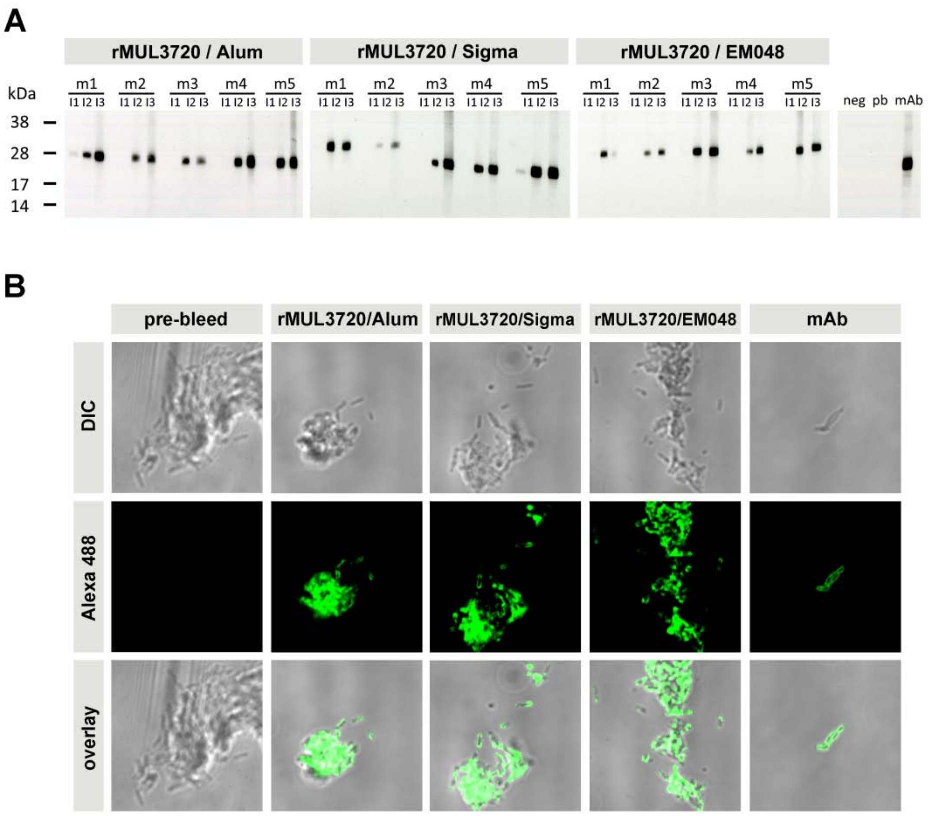

Figure S2: Cross reactivity of immune sera with *M. ulcerans*.

(A) Groups of five BALB/c mice (m1 – m5) were immunized three times in three week intervals with 20 µg of rMUL3720 formulated with Alum, Sigma Adjuvant or EM048. Serum after every immunization (I1, I2 and I3) was analysed by Western blotting on *M. ulcerans* lysate. Monoclonal anti-MUL_3720 antibody (mAb) served as positive control, pre-bleed (pb) serum or no primary antibody (nc) as negative controls. (B) Sera from three weeks after the third immunization with rMUL3720 and indicated adjuvant were used for indirect immunofluorescence staining on paraffin embedded *M. ulcerans* bacteria with an Alexa488 labelled secondary antibody. Pre-bleed serum did not stain the bacteria. Sera of immunized mice (a mix of sera from five individual mice per group) did reveal surface staining similar to the staining achieved with anti-MUL_3720 monoclonal antibody (mAb).
