# Supplementary material for: Vaccination with the Surface Proteins MUL_2232 and MUL_3720 of Mycobacterium ulcerans Induces Antibodies but Fails to Provide Protection against Buruli Ulcer
Source: PLoS Negl Trop Dis. 2016 Feb 5;10(2):e0004431. doi: 10.1371/journal.pntd.0004431 (PMC4746116; doi:10.1371/journal.pntd.0004431)
Supplement: S3 Fig — Groups of six BALB/c mice were immunized twice with 20 μg of rMUL2232/EM048, PBS/EM048 or PBS alone as infection control. Three weeks after the last immunization mice were challenged with M. ulcerans (inoculum) into the left hind foot pad. Infection was followed by measuring foot pad thickness with a caliper (A1) until mice were euthanized at day 42 after infection. Depicted is the mean foot pad thickness (diamond/dot) ± standard deviation of the differently immunized groups. (A2) Bacterial load in infected foot pads was determined by qPCR for five mice per group. Depicted are individual measurements as genome copies per foot pad, the mean (line) ± standard deviation. (PDF) [file pntd.0004431.s003.pdf]

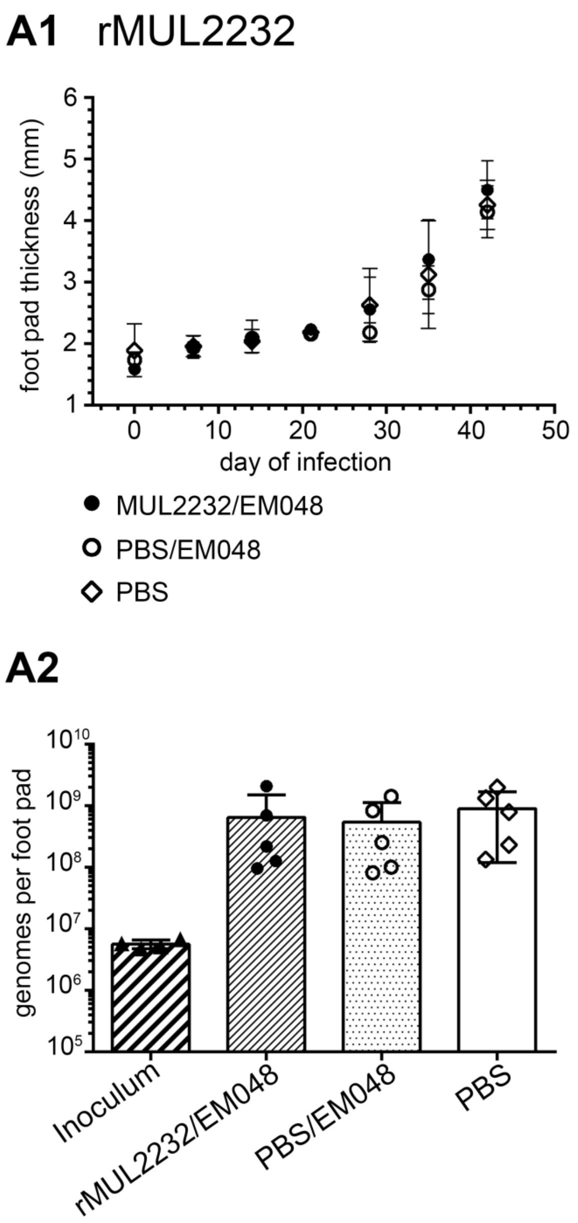

Figure S3: Evaluation of the protective potential of immunization with rMUL2232/EM048 formulation in a *M. ulcerans* infection mouse model.

Groups of six BALB/c mice were immunized twice with 20 µg of rMUL2232/EM048, PBS/EM048 or PBS alone as infection control. Three weeks after the last immunization mice were challenged with *M. ulcerans* (inoculum) into the left hind foot pad. Infection was followed by measuring foot pad thickness with a caliper (A1) until mice were euthanized at day 42 after infection. Depicted is the mean foot pad thickness (diamond/dot) ± standard deviation of the differently immunized groups.

(A2) Bacterial load in infected foot pads was determined by qPCR for five mice per group. Depicted are individual measurements as genome copies per foot pad, the mean (line) ± standard deviation.
