# Supplementary material for: Vaccination with the Surface Proteins MUL_2232 and MUL_3720 of Mycobacterium ulcerans Induces Antibodies but Fails to Provide Protection against Buruli Ulcer
Source: PLoS Negl Trop Dis. 2016 Feb 5;10(2):e0004431. doi: 10.1371/journal.pntd.0004431 (PMC4746116; doi:10.1371/journal.pntd.0004431)
Supplement: S4 Fig — Groups of eight BALB/c mice were immunized twice with 20 μg of rMUL3720/EM048 or PBS only as infection control. Serum prior to infection with M. ulcerans was analysed by Western blotting on M.ulcerans lysate. Monoclonal anti-MUL_3720 antibody (mAb) served as positive control, pre-bleed (pb) serum or no primary antibody (neg) as negative controls. C1 and C2 each represent a mix of sera of eight mice immunized with PBS only. (PDF) [file pntd.0004431.s004.pdf]

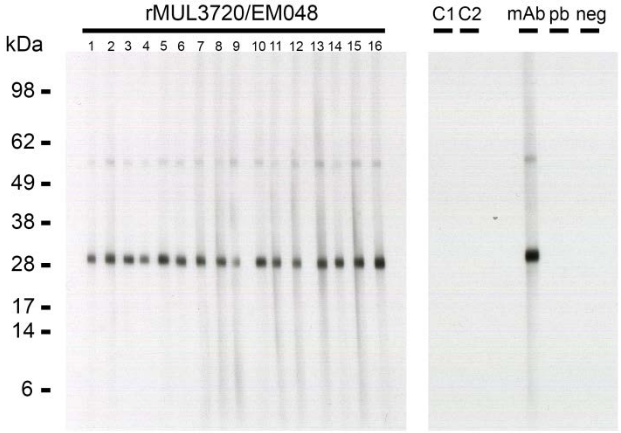

Figure S4: Reactivity of immune sera on *M. ulcerans* lysate.

Groups of eight BALB/c mice were immunized twice with 20 µg of rMUL3720/EM048 or PBS only as infection control. Serum prior to infection with *M. ulcerans* was analysed by Western blotting on *M.ulcerans* lysate. Monoclonal anti-MUL_3720 antibody (mAb) served as positive control, pre-bleed (pb) serum or no primary antibody (neg) as negative controls. C1 and C2 each represent a mix of sera of eight mice immunized with PBS only.
